# A novel target enrichment strategy in next-generation sequencing through 7-deaza-dGTP-resistant enzymatic digestion

**DOI:** 10.1186/s13104-020-05292-y

**Published:** 2020-09-18

**Authors:** Peng Peng, Yanjuan Xu, Adrian M. Di Bisceglie, Xiaofeng Fan

**Affiliations:** 1 Division of Gastroenterology & Hepatology, Department of Internal Medicine, Saint Louis University School of Medicine, St. Louis, MO 63104 USA; 2 Saint Louis University Liver Center, Saint Louis University School of Medicine, St. Louis, MO 63104 USA; 3 Wuhan Pulmonary Hospital, Wuhan, 430030 Hubei China

**Keywords:** Next-generation sequencing, Target enrichment, 7-deaza-2′-deoxyguanosine 5′-triphosphate, Hepatitis B virus

## Abstract

**Objective:**

Owing to the overwhelming dominance of human and commensal microbe sequences, low efficiency is a major concern in clinical viral sequencing using next-generation sequencing. DNA composed of 7-deaza-2′-deoxyguanosine 5′-triphosphate (c^7^dGTP), an analog of deoxyguanosine triphosphate (dGTP), is resistant to selective restriction enzymes. This characteristic has been utilized to develop a novel strategy for target enrichment in next-generation sequencing.

**Results:**

The new enrichment strategy is named target enrichment via enzymatic digestion in next-generation sequencing (TEEDseq). It combined 7-deaza-2′-deoxyguanosine 5′-triphosphate (c^7^dGTP)-involved primer extension, splinter-assisted intracellular cyclization, c^7^dGTP)-resistant enzymatic digestion, and two-phase rolling cycle amplification. We first estimated c7dGTP for its efficiency in PCR amplification and its resistance to three restriction enzymes, AluI, HaeIII, and HpyCH4V. We then evaluated TEEDseq using a serum sample spiked with a 1311-bp hepatitis B virus (HBV) fragment. TEEDseq achieved an HBV on-target rate of 3.31 ± 0.39%, which was equivalent to 454× the enrichment of direct Illumina sequencing. Therefore, the current study has provided a concept proof for TEEDseq as an alternative option for clinical viral sequencing that requires an enrichment in next-generation sequencing.

## Introduction

In current clinical viral genome sequencing, next-generation sequencing (NGS) is a frequent choice that provides an unbiased high resolution of mutation profile in a genome-wide manner [1]. Because of an overwhelming dominance of human genetic content in clinical specimens, a major limitation of this approach is its low efficiency, which is rarely higher than 1% of viral sequencing reads in NGS output [1]. Among numerous virus-enriched methods, capture sequencing, employing a hybridization step after NGS library construction, comes out as the most efficient strategy to enrich viral sequences [1]. However, this strategy is associated with a dramatic cost increase as it requires the synthesis of expensive biotin labeled virus-specific probes (baits) and streptavidin beads [2]. The inclusion of such a hybridization step after initial library preparation also makes the entire NGS pipeline a lengthy procedure. Most human viruses, such as hepatitis B virus (HBV), hepatitis C virus (HCV), HIV, and coronavirus, have a genome less than 30 kb in size. If the viral on-target rate consistently exceeds 1%, current NGS approach is actually powerful enough to satisfy clinical and research needs. For instance, a 1% HCV on-target rate in 5 million of 2 × 150 bp paired ended reads give a depth at 1562×, which already crosses a saturation point (1100×) for HCV viral population dissection with a mutation frequency resolved at 1% [3]. To achieve this goal, we provide an alternative option for viral sequence enrichment that does not require a probe-based hybridization step. Our method, named NGS with target enrichment via enzymatic digestion (TEEDseq), is dependent on 7-deaza-2′-deoxyguanosine 5′-triphosphate (c^7^dGTP), an analog of deoxyguanosine triphosphate (dGTP). Due to its ability to relax DNA secondary structure, c^7^dGTP is widely used in PCR and Sanger sequencing [4, 5]. DNA molecules composed of c^7^dGTP show steric alteration that is resistant to some restriction enzymes with the recognition motifs containing guanosine [6]. This unique characteristic of c^7^dGTP is used to accomplish the enrichment of a sequencing target.

## Main text

### Materials and methods

PCR amplification efficiency using c^7^dGTP was first estimated using an HBV plasmid as the template [7]. A 30-cycle PCR was done in a 50 µL reaction containing 1× Q5 DNA polymerase buffer, 0.8 mM dNTPs, each 0.4 µM of primers HBVF1 and HBVR1 (Table 1), and 1 unit of Q5 DNA polymerase [New England Biolabs (NEB), Ipswich, MA]. In the parallel reaction, dGTPs was completely replaced by c^7^dGTP (Roche Molecular Systems, Madison, WI). After the purification with QIAquick PCR Purification Kit (Qiagen, Valencia, CA), the PCR product was quantitated in NanoDrop 2000 Spectrophotometer (Thermo Fisher Scientific, Waltham, MA).

**Table 1:** List of the oligonucleotides used in the study

Oligonucleotide	Polarity	Sequence (5′ → 3′)	Position	Product size/note
HBVF1	Sense	actctctcgtccccttctcc	1479–1498	504 bp
HBVR1	Antisense	tgacggaaggaaagaagtcag	1962–1982	
HBVR1^p^	Antisense	^p^tgacggaaggaaagaagtcag	1962–1982	
HBVF2	Sense	ccttctccgtctgccgttc	1491–1509	488 bp
HBVR2	Antisense	ggaaggaaagaagtcagaaggc	1957–1978	
HBVF3	Sense	aacaggctttcactttctcgc	1082–1102	1311 bp
HBVR3	Antisense	cgagggagttcttcttctaggg	2371–2392	
HBVR4	Antisense	^p^tccacactccgaaagagacc	2257–2276	
Splinter	NA	nnnnnnaggtgtgtSp3	To facilitate intramolecular ligation	
HBVR5	Antisense	tgtgtg*g*a	Target-specific RCA	
C28	NA	Sp18nnn*n*n	Non-specific RCA	

Position is according to the full-length HBV genome under GenBank accession number AB241115. Star donated phosphorothioate bonds to resist exonuclease activity of phi29 DNA polymerase. P in superscript indicated the modification of phosphate at the 5′ ends. C28 is the primer to eliminate primer-mediated artifacts from phi29 DNA polymerase-based multiple displacement amplification in our previous study [11]. Sp3, C3 spacer to block self-ligation of the splinter; Sp18, C18 spacer; NA, not applicable. All oligonucleotides were ordered from the Integrated DNA Technologies, Coralville, IA

Next, we tested the resistance of c^7^dGTP to restriction enzymes. The above PCR was repeated with primer HBVR1^p^ containing a phosphate at 5′ end. PCR product was purified and digested by Lambda exonuclease (NEB) that favored the digestion of 5′ phosphate strand [8]. Consequently, both single-strand DNA (ssDNA) and double-strand DNA (dsDNA) were generated and used for the digestion of three restriction enzymes (AluI, HaeIII, and HpyCH4V) (NEB) that were carefully selected based on their recognition motifs and buffer compatibility. The experiment was repeated with PCR amplicon generated using c^7^dGTP. Since c^7^dGTP was difficult to stain using ethidium bromide [9], PCR with a high cycle number (n = 35) was conducted using an aliquot of 2 µL enzyme reaction with primers HBVF2 and HBVR2 (Table 1).

Finally, TEEDseq was evaluated using a healthy donor serum sample spiked with a 1311-bp HBV PCR fragment at a concentration of 1 × 10^6^ copies/mL, mimicking a concentration from viruses like HCV and HBV (Table 1) [10]. Total DNA was extracted from 0.5 mL of serum by QIAamp MinElute ccfDNA Kit (Qiagen, Valencia, CA) and eluted into 20 µL Tris buffer. Entire 20 µL extracted DNA was used for 5-cycle primer extension under 1 unit of Q5 DNA polymerase, 0.4 µM primer HBVR4 (Table 1), and 0.8 mM of dNTPs in which dGTP was completely replaced by c^7^dGTP. The reaction was purified using MinElute PCR Purification Kit (Qiagen) and eluted into 20 µL Tris buffer, followed by ligation in 30 µL reaction consisting of 10 U T4 DNA ligase and 0.5 µM of the splinter at 14 °C overnight. After heat inactivation, the enzyme complex (AluI 10 U, HeaIII 10 U, HpyCH4V 5U, Exonuclease I 50 U, and Exonuclease III 20 U) was added to bring the reaction up to 45 µL volume in 1× CutSmart buffer. 3 h after the incubation at 37 °C, the reaction was heat-inactivated and used for RCA with 1 µM target-specific primer HBVR5 (Table 1) at 30 °C for the first 12 h and then 4 h at 28 °C with 80 µM of C28 primer (Table 1). The final product was around 12 kb in size with an average yield of 1.8 µg after purification using QIAprep Spin Miniprep Kit (Qiagen). The product was subjected to Illumina sequencing (1 × 250 nt single-end read), followed by data analysis as we previously described [11, 12]. We tested four options: full TEEDseq protocol (a), TEEDseq with the omission of three restriction enzyme (b), direct sequencing using Illumina Nextera Flex for plasma/serum kit (c), and full TEEDseq using the same serum sample without the spike-in of the HBV fragment (d). Each option was set for three technical replicates.

## Results

PCR using c^7^dGTP showed a weak band in ethidium bromide (EB)-stained gel (Additional file 1: Figure S1A), which was consistent with the previous report that c^7^dGTP was hardly stained using EB [9]. However, PCR quantification revealed a slightly lower yield with c^7^dGTP (Additional file 1: Figure S1B). This slight drop in PCR yield may also be attributed to the nature of c^7^dGTP rather than an authentic decrease. Hence, PCR with c^7^dGTP had similar efficiency to that using regular dGTP.

In the estimation of c^7^dGTP’s resistance to restriction enzymes, the amplicon had three AluI sites, one HaeIII site, and four HpyCH4V sites. While all three enzymes had a complete digestion of dsDNA, HpyCH4V cut both ssDNA and dsDNA (Additional file 1: Figure S1C). In comparison to dGTP, c^7^dGTP showed strong bands, suggesting a resistance to digestion. The combination of all three enzymes resulted in almost a complete digestion of both ssDNA and dsDNA, as indicated by a much weaker band (Additional file 1: Figure S1D). These experiments have demonstrated that dc7GTP is resistant to individual and combinatorial digestion of AluI, HaeIII, and HpyCH4V.

**Fig. 1:**
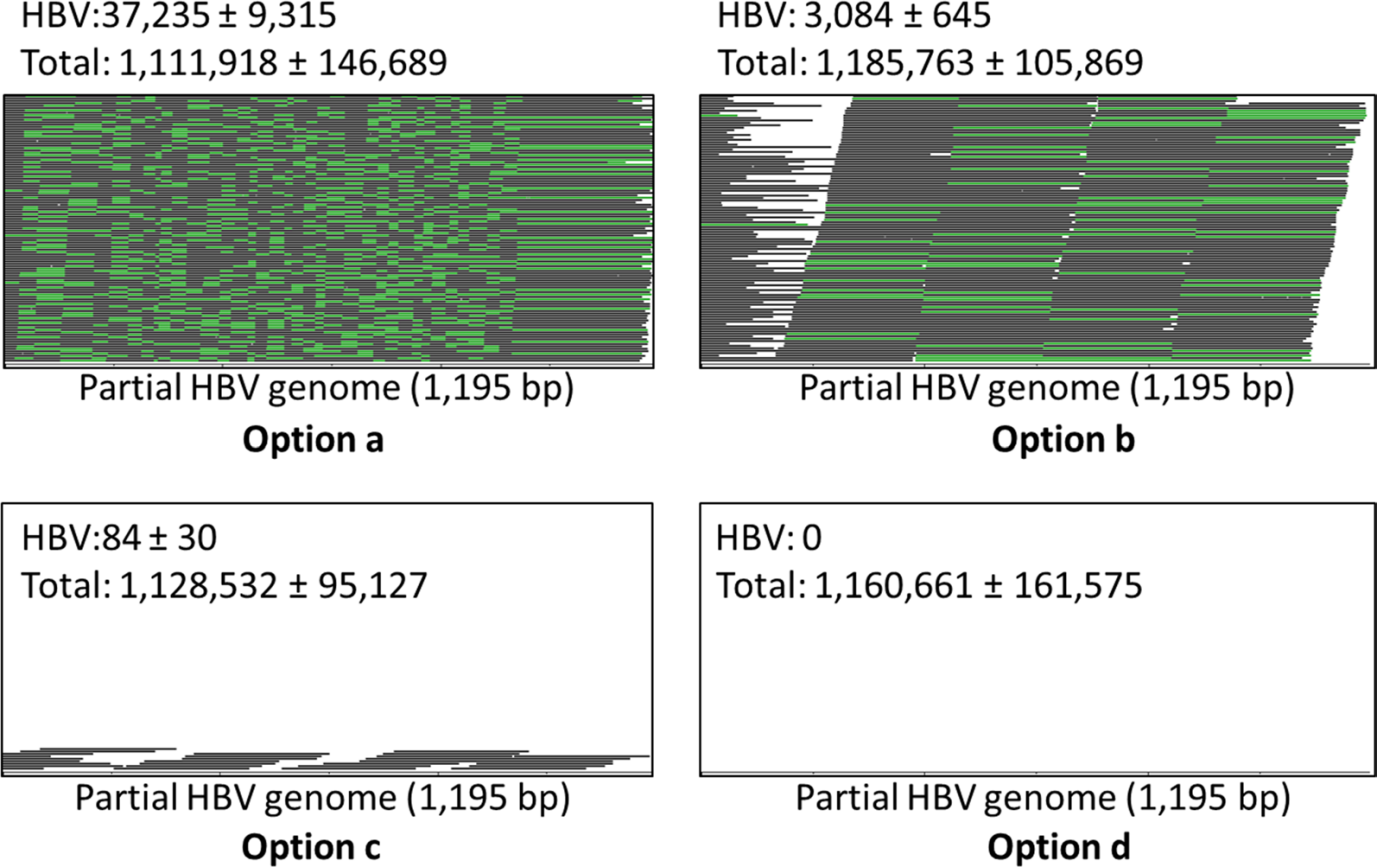
HBV-specific read mapping among four options. Read-alignment on 1195-bp HBV genome sequence from the HBVR4 priming site was viewed in bam file using BamView [13]. Reads with matching start and end positions were collapsed into one line and are shown in green. Option a, b, and c used a serum sample spiked with 1311-bp HBV fragment. Option d had no HBV fragment spiked in the serum and served as a control. Each option was shown with the numbers (average and standard derivation) of HBV-mapped and total reads from three technical replicates after the quality control

After read quality control [11, 12], one million of total reads had HBV-mapped reads at 33,153 ± 3900 (3.31 ± 0.39%), 2638 ± 750 (0.26 ± 0.07%), 73 ± 21 (0.007 ± 0.0002%), and zero for options a, b, c, and d, respectively (Fig. 1). TEEDseq reached an enrichment 454× that of direct sequencing (option c). The recovery of HBV-mapped reads was 12.6 times higher in option a than in option b, illustrating the pivotal role of the three restriction enzymes. Using HiCUP [14], these enzymes together have 36,535,384 cuts (AluI 13,085,321; HaeIII 8,582,925; HpyCH4V 14,867,138) on the human genome (building GRCh38). Their combination with exonucleases efficiently digested non-target background sequences.

## Discussion

**Fig. 2:**
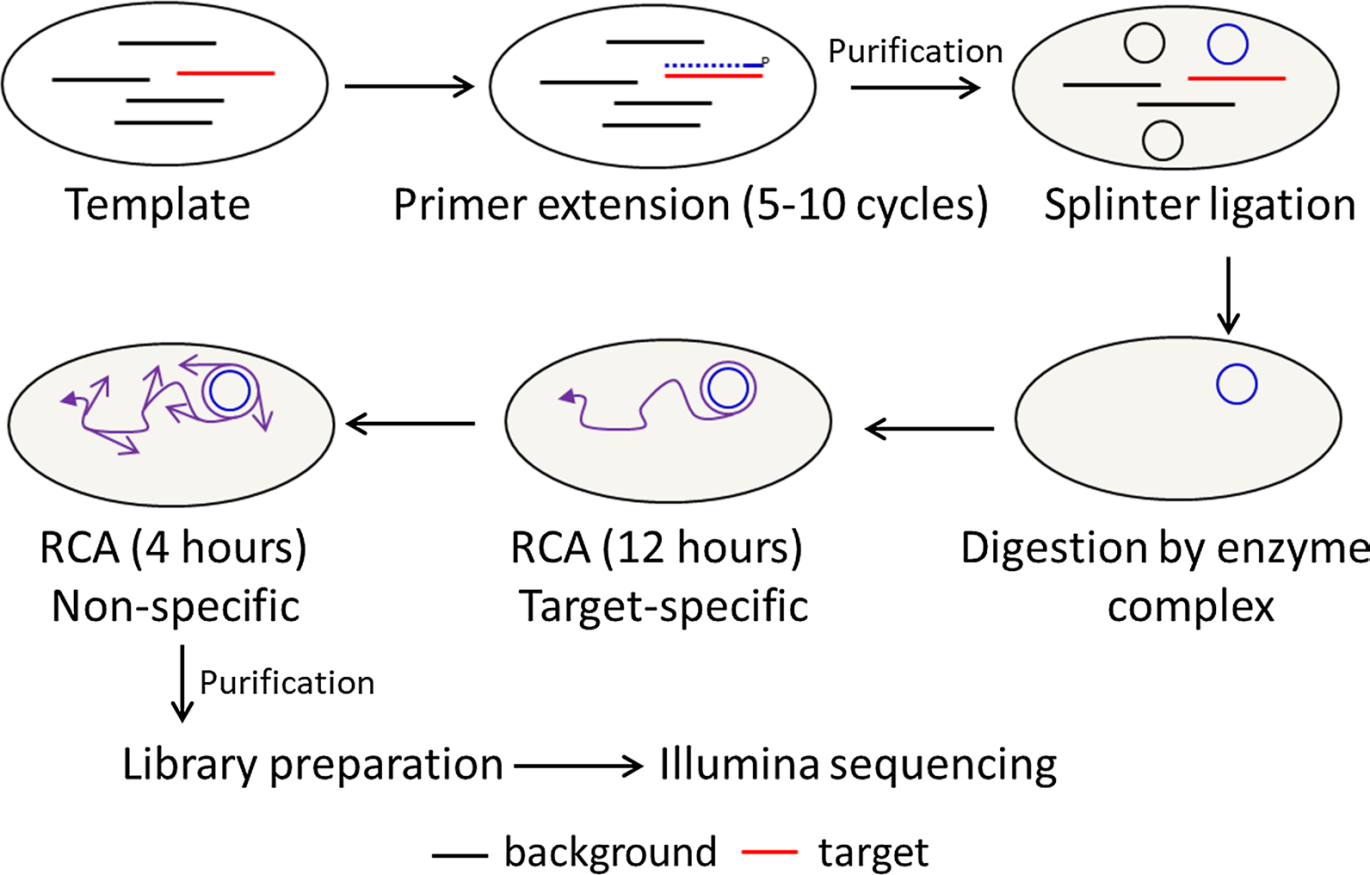
The working flow of TEEDseq. Note that ligation, digestion, and RCA (grey-filled cycles) are placed in the same tube in a sequential manner. RCA, rolling cycling amplification

Our method consists of four steps: primer extension, splinter ligation, enzymatic digestion, and rolling circle amplification (RCA) (Fig. 2). Using a serum sample spiked with a partial HBV genome (1311 bp), TEEDseq achieved a 3.31% mapping rate. Under a probe-based hybridization strategy, genome-wide HBV capture sequencing does not necessarily have a high on-target rate, for instance, < 1% in a recent report [15]. Off-target effect may come from non-specific priming since there is significant micro-homology between HBV and the human genome [16]. A more rigid primer design and conditions for primer extension could further enhance the enrichment.

In addition, TEEDseq has several technical features worthy of attention. Serum DNA is regarded as a low-biomass sample. Its low DNA concentration, 435 ng/mL in the current study, naturally favors intracellular ligation that can be further facilitated using a splinter. Because intermolecular ligation is suppressed at a low DNA concentration, high concentration of templates, such as DNA extracted from tissue samples, need to be diluted prior to the ligation [17]. Second, we applied two-phase RCA amplification, target-specific and non-specific. The short incubation of non-specific RCA suppresses the amplification of contaminated sequences in the reagents, as observed in our recent studies [18, 19]. Third, TEEDseq requires purification after primer extension. Afterwards, ligation, digestion, and RCA do not need purification because all enzymes have optimal activity in the CutSmart buffer (NEB). Therefore, these reactions can be conducted successively in the same tube. Finally, phi29 DNA polymerase used in RCA has a strong stand-displacement activity. This activity results in a hyperbranched structure of the final product that usually has a large size more than 10 kb [20]. Therefore, the final product can be directly used for fragmentation in NGS library preparation without the need of additional procedure, such as concatemerization. Taken together, our experiment, using a partial HBV genome (1311 bp) spiked in a serum sample, provides concept evidence that TEEDseq is a simple and cost-effective method for target enrichment in NGS. By using multiple primers to cover target genomes in primer extension, it can be applied to clinical viral sequencing as well as human genomic research.

## Limitations

The current study is merely a proof of principle for TEEDseq. It remains to be improved toward a simple experimental method. For instance, time for the steps of ligation and RCA may be shortened. In addition, the efficiency and sensitivity of TEEDseq need to be further evaluated in clinical specimens.

## Supplementary information

**Additional file 1: Figure S1**. Characteristics of c7dGTP-involved PCR amplification and digestion. PCR product with c7dGTP was hard to stain by EB (A) but having similar yield to dGTP (B). All three enzymes gave complete digestion of dsDNA generated from PCR using dGTP (C) but not c7dGTP, as examined via 35-cycle of PCR after digestion (D).

## Data Availability

Sequence data in fastq format was deposited in the NCBI Sequence Read Archive (SRA) under BioProject ID: PRJNA626058. All other data generated or analyzed during this study are included in this published article [and its additional files].

## Abbreviations

TEEDseq: Target enrichment via enzymatic digestion in next generation sequencing
NGS: Next-generation sequencing
c^7^dGTP: 7-Deaza-2′-deoxyguanosine 5′-triphosphate
dGTP: Deoxyguanosine triphosphate
RCA: Rolling cycling amplification
EB: Ethidium bromide
ssDNA: Single-strand DNA
dsDNA: Double-strand DNA
HBV: Hepatitis B virus
HCV: Hepatitis C virus

## References

[1] Houldcroft CJ, Beale MA, Breuer J (2017). Clinical and biological insights from viral genome sequencing. Nat Rev Microbiol.

[2] Briese T, Kapoor A, Mishra N, Jain K, Kumar A, Jabado OJ (2015). Virome capture sequencing enables sensitive viral diagnosis and comprehensive virome analysis. MBio.

[3] Wang W, Zhang X, Xu Y, Weinstock GM, Di Bisceglie AM, Fan X (2014). High-resolution quantification of hepatitis C virus genome-wide mutation load and its correlation with the outcome of peginterferon-alpha2a and ribavirin combination therapy. PLoS ONE.

[4] McConlogue L, Brow MA, Innis MA (1998). Structure-independent DNA amplification by PCR using 7-deaza-2′-deoxyguanosine. Nucleic Acids Res.

[5] Motz M, Pääbo S, Kilger C (2000). Improved cycle sequencing of GC-rich templates by a combination of nucleotide analogs. Biotechniques.

[6] Grime SK, Martin RL, Holaway BL (1991). Inhibition of restriction enzyme cleavage of DNA modified with 7-deaza-dGTP. Nucleic Acids Res.

[7] Valenzuela P, Rall L, Zaldivar M, Quiroga M, Gray P, Rutter W (1980). The nucleotide sequence of the hepatitis B viral genome and the identification of the major viral genes. ICN-UCLA Symp Mol Cell Biol..

[8] Civit L, Fragoso A, O’Sullivan CK (2012). Evaluation of techniques for generation of single-stranded DNA for quantitative detection. Anal Biochem.

[9] Weiss J, Zucht HD, Forssmann WG (1994). Amplification of gene fragments with very high G/C content: c7dGTP and the problem of visualizing the amplification products. PCR Methods Appl..

[10] Easterbrook PJ, Roberts T, Sands A, Peeling R (2017). Diagnosis of viral hepatitis. Curr Opin HIV AIDS.

[11] Wang W, Ren Y, Xu Y, Crosby S, Di Bisceglie AM, Fan X (2017). Template-dependent multiple displacement amplification applied to profile human circulating RNA. Biotechniques.

[12] Gorse GJ, Gira B, Patela GB, Fan X (2017). Interpatient mutation spectrum of human coronavirus-OC43 revealed by illumina sequencing. J Med Virol.

[13] Carver T, Harris SR, Otto TD, Berriman M, Parkhill J, McQuillan JA (2013). BamView: visualizing and interpretation of next-generation sequencing read alignments. Brief Bioinform.

[14] Wingett S, Ewels P, Furlan-Magaril M, Nagano T, Schoenfelder S, Fraser P (2015). HiCUP: pipeline for mapping and processing Hi-C data. F1000Res..

[15] Zhao LH, Liu X, Yan HX, Li WY, Zeng X, Yang Y (2016). Genomic and oncogenic preference of HBV integration in hepatocellular carcinoma. Nat Commun..

[16] Lin S (2016). Analysis of the complexity of HBV-host junction sequences in patients with HBV-related hepatocellular carcinoma.

[17] Mason WS, Gill US, Litwin S, Zhou Y, Peri S, Pop O (2016). HBV DNA integration and clonal hepatocyte expansion in chronic hepatitis B patients considered immune tolerant. Gastroenterology.

[18] Li G, Zhou Z, Yao L, Xu Y, Wang L, Fan X (2019). Full annotation of serum virome in Chinese blood donors with elevated alanine aminotransferase levels. Transfusion.

[19] Ren Y, Xu Y, Lee WM, Di Bisceglie AM, Fan X (2020). In-depth serum virome analysis in acute liver failure patients with indeterminate etiology. Arch Virol.

[20] Binga EK, Lasken RS, Neufeld JD (2008). Something from (almost) nothing: the impact of multiple displacement amplification on microbial ecology. ISME J.

