# Supplementary material for: A novel target enrichment strategy in next-generation sequencing through 7-deaza-dGTP-resistant enzymatic digestion
Source: BMC Res Notes. 2020 Sep 18;13:445. doi: 10.1186/s13104-020-05292-y (PMC7499927; doi:10.1186/s13104-020-05292-y)
Supplement: Supplementary file 1 — Additional file 1: Figure S1. Characteristics of c7dGTP-involved PCR amplification and digestion. PCR product with c7dGTP was hard to stain by EB (A) but having similar yield to dGTP (B). All three enzymes gave complete digestion of dsDNA generated from PCR using dGTP (C) but not c7dGTP, as examined via 35-cycle of PCR after digestion (D). [file 13104_2020_5292_MOESM1_ESM.pdf]

Title: **A Novel Target Enrichment Strategy in Next-generation Sequencing through 7-deaza-dGTP-resistant Enzymatic Digestion**

Supplementary Material

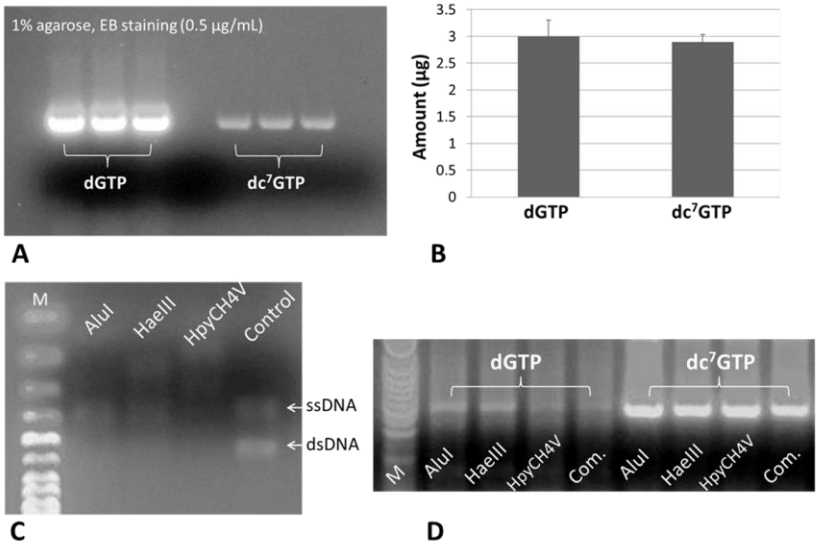

Figure S1. Characteristics of c7dGTP-involved PCR amplification and digestion. PCR product with c7dGTP was hard to stain by EB (A) but having similar yield to dGTP (B). All three enzymes gave complete digestion of dsDNA generated from PCR using dGTP (C) but not c7dGTP, as examined via 35-cycle of PCR after digestion (D)
